# *In Vitro* Antimicrobial and Antiprotozoal Activities, Phytochemical Screening and Heavy Metals Toxicity of Different Parts of *Ballota nigra*

**DOI:** 10.1155/2014/321803

**Published:** 2014-06-26

**Authors:** Najeeb Ullah, Ijaz Ahmad, Sultan Ayaz

**Affiliations:** ^1^Department of Chemistry, Kohat University of Science & Technology, Kohat, Khyber Pakhtunkhwa 26000, Pakistan; ^2^Department of Zoology, Kohat University of Science & Technology, Kohat, Khyber Pakhtunkhwa 26000, Pakistan

## Abstract

The study was done to assess the phytochemicals (flavonoids, terpenoids, saponins, tannin, alkaloids, and phenol) in different parts (root, stem, and leaves) of * Ballota nigra *and correlated it to inhibition of microbes (bacteria and fungi), protozoan (Leishmania), and heavy metals toxicity evaluation. In root and stem flavonoids, terpenes and phenols were present in ethanol, chloroform, and ethyl acetate soluble fraction; these were found to be the most active inhibiting fractions against all the tested strains of bacteria, fungi, and leishmania. While in leaves flavonoids, terpenes, and phenols were present in ethanol, chloroform, and * n-*butanol fractions which were the most active fractions against both types of microbes and protozoan (leishmania) in* in vitro* study. Ethanol and chloroform fractions show maximum inhibition against * Escherichia coli* (17 mm). The phytochemical and biological screenings were correlated with the presence of heavy metals in selected plant* Ballota nigra. *Cr was found above permissible value (above 1.5 mg/kg) in all parts of the plant. Ni was above WHO limit in * B. nigra *root and leaves (3.35 ± 1.20 mg/kg and 5.09 ± 0.47 mg/kg, respectively). Fe was above permissible value in all parts of * B. nigra * (above 20 mg/kg). Cd was above permissible value in all parts of the plant (above 0.3 mg/kg). Pb was above WHO limit (above 2 mg/kg) in all parts of * Ballota nigra*.

## 1. Introduction

Different parts (fruit, flower, leaf, stem, root, and twigs) of medicinal plants of wide variety are used as drugs because of medicinal properties. Some of these drugs are used in crude form by local hakims and healers. Some are used in smaller quantities, while other drugs used in raw form are gathered in large quantities for trading in the market [1]. In developing countries most of the people use traditional medicines for healing purposes obtained from plants or herbs. Antibiotics have some common side effects on host which include allergic reactions, immune suppression, and hypersensitivity. All these screening studies show that herbal extracts have antimicrobial activity [2].

Mericli et al. 1988 studied that* Ballota acetabulosa* is used in folk medicines [3]. Saltan Çitoğlu et al. 2003 performed experiment on antimicrobial activities of 16* Ballota* species growing in Turkey [4]. Crude ethanolic extract was used against both gram positive and gram negative bacterial strains in* in vitro *study. The crude ethanolic extract was also used against different fungal strains (*C. krusei, C. Glabrata, *and* C. albicans*). Both of the antimicrobial activities were performed by disc diffusion method. The results show that* Ballota* species were good antimicrobial and excellent antifungal agents [5].

Microorganisms can produce many different kinds of toxins in different stages of their life cycle. These toxins then act as antigens for human, plants, and other animals and cause serious problems and infections. These organisms are commonly spread through blood and lymphatic system [6].

Leishmaniasis is a disease caused by a trypanosomatid parasite belongs to Protozoa family. Genus of this parasite is* Leishmania*. Its transmitting source is a female sand fly Phlebotomine by biting humans. There are three different forms of leishmaniasis, that is, cutaneous, visceral, and mucocutaneous leishmaniasis. Out of these three types, cutaneous leishmaniasis is deadly. In these days ten million people suffer from cutaneous leishmaniasis. One in twenty individuals worldwide is suffered from cutaneous leishmaniasis [7].

Medicinal plants contain useful active substances that can be used for healing purposes either in their pure form or as precursors to synthesize new useful drugs. The importance of these medicinal plants depends upon the presence of phytochemical substances active against different microbes [8].

Plants accumulate different heavy metals in different concentration. Excessive intake of herbal products may cause excessive intake of heavy metals which may result in serious complications like accumulative poisoning, damaged nervous system, cancer, and ultimate death [9]. Contamination by heavy metals like cadmium, copper, lead, and nickel, when accumulated in plants above permissible value, causes environmental pollution and can cause serious health complications. Therefore users should be aware of permissible limits of heavy metals [10]. Since animals are sensitive to heavy metals concentration, as a result it produces different illnesses. Like animals, plants are also sensitive to heavy metals concentration [11]. Human activities play a major role in the increase of heavy metals concentration in environment. During the last few decades in the environment heavy metals level has been seriously increased mainly due to human activities. Therefore, it is very much necessary to determine the heavy metals contamination in environment, very precisely and rapidly, particularly to toxic heavy metals [12].

## 2. Materials and Methods

### 2.1. Collection and Drying of Plant Materials

Sufficient quantity of* Ballota nigra *was collected from Latamber, District Karak, Khyber Pakhtunkhwa, and was identified by Nisar Ahmad, Lecturer, Department of Botany, Kohat University of Science & Technology, Kohat, Pakistan. The samples were properly rinsed with distilled water to eliminate dirt, dust, and other possible parasites and then were shade-dried at 25–30°C. The dried parts (root, stem, and leaves) were powdered unconnectedly and then stored in clean, dried plastic bags for further processing.

### 2.2. Extraction Procedure

The dried root, stem, and leaves of* weight* 2 kg each of* Ballota nigra* were taken and soaked in ethanol for 15 days separately and were extracted at room temperature in the same solvent and then filtered. The filtrates were evaporated under reduced pressure by vacuum rotary evaporator at 35°C to give crude extracts (210 gm). These extracts were dried and weighed, which were further suspended in water and partitioned successively with* n-*hexane, chloroform, ethyl acetate, and* n-*butanol to obtain their soluble fractions. Weight of each fraction was* n-*hexane (23 gm), chloroform (42 gm), ethyl acetate (25 gm),* n-*butanol (30 gm), and aqueous fraction (60 gm).

### 2.3. Antimicrobial Assay

In this study, extracts and various fractions of* Ballota nigra *were evaluated for antimicrobial activities against gram positive and gram negative bacteria and against fungi. The six bacterial strains* Escherichia coli, Staphylococcus aureus, Proteus mirabilis, Klebsiella pneumoniae, Enterococcus faecalis, *and* Salmonella typhi* were used and four fungal strains* Aspergillus niger, Aspergillus flavus, Aspergillus fumigatus, *and* Fusarium solani* were tested.

### 2.4. Preparation of the Test Compound

Dimethyl sulfoxide oxide (DMSO) solutions of extracts and fractions at the same concentration of 2 *μ*g/*μ*L were prepared. DMSO was used as a solvent, because it does not confirm any inhibitory activity against bacteria and fungi [13, 14]. In separate conical flasks, solution of concentration of 28 g/L nutrient agar was prepared. Media solution, petri plates, and borer were sterilized for 15 minutes at 1.5 pound pressure at 121°C in autoclave. Nutrient agar media was poured in Petri plates in laminar flow and allowed to solidify for 20 minutes.

### 2.5. Antibacterial Bioassay

The antibacterial bioassay was made by agar well diffusion method, by measuring the zone of inhibition against the test microorganisms by using Asghari method with a little modification [15].

### 2.6. Well Assay Method

Wells of 6 mm were dug in media by using sterile plastic borer. Each well was given a specific number. Bacterial culture was inoculated/streaked on the surface of the solidified media. Stock solutions of crude extracts and fractions were added into respective wells. The zones of inhibition were measured after 24 hours of incubation at 37°C in incubator. Amoxicillin (5 *μ*g/*μ*L) and levofloxacin (5 *μ*g/*μ*L) were used as standards as positive control while DMSO was used as a negative control. The zones of inhibition of crude extracts,* n-*hexane, chloroform, ethyl acetate,* n-*butanol, and aqueous fractions were compared with zones of inhibition of standard drugs amoxicillin and levofloxacin. The total of growth in each well was measured.

### 2.7. Antifungal Bioassay

The antifungal bioassay was determined by agar tube dilution method by using Rehman et al. (2001) method with some modifications [16].

### 2.8. Agar Tube Dilution Method

Four fungal strains, that is,* Aspergillus flavus, Fusarium solani, Aspergillus fumigatus, *and* Aspergillus niger*, were used for antifungal activities. To refresh fungal strains, nutrient broth solution of concentration 13 g/L in distilled water was prepared. Nutrient Broth media was sterilized in autoclave, and distributed equally to four flasks of 250 mL each. To each flask fungal colonies were inoculated separately. These flasks were then placed in incubator at 30°C for 3 days to refresh fungal strains. In a 1-liter conical flask 28 grams of nutrient agar was taken and dissolved in 1 L of distilled water. The flask was sterilized in autoclave at 121°C for 15 minutes at 1.5 pounds pressure. An antibiotic, Clotrimazole, was taken as a positive control standard and dissolved in (2 *μ*g/*μ*L) distilled water, while DMSO (6 *μ*L/disc) was taken as negative control. About 9 mL of medium was added to clean, dry, and sterilized test tubes. Solutions of crude extracts and subfractions were prepared each of 2 *μ*g/*μ*L concentration. One mL of sample (2 *μ*g/*μ*L) was also added to each test tube; the test tube was kept in inclined position to make a slant. The same process was repeated for all the test tubes. After cooling and solidifying, the fungi inoculums suspension was spread over the agar medium homogeneously using sterile cotton swabs. After that the test tubes were kept in incubator for 3 daysat 30°C. After 3 days the fungal growth was observed in each test tube.

### 2.9. Antileishmanial Bioassay

*In vitro* antileishmanial activity of crude extracts and other fractions of* Ballota nigra* were evaluated. For this purpose the specimens were collected from Latamber, Karak, Khyber Pakhtunkhwa, Pakistan, from many patients having cutaneous leishmaniasis. The area of infection and the adjacent normal looking skin around the infection were washed and sterilized with ethanol. With the help of lancet or sterilized surgical blade skin cuts were made till blood oozes out from the infected part in one periphery. In the margin of scratch, blood is collected and stored in eppendorf containing 0.9% saline solution. A miniature quantity of sample was applied on slide and then observed under the microscope to test out nonflagellated amastigote, whereas in other parts buffer solution of pH 7.2 was mixed and placed in incubator for further process. The sample, in which amastigote was observed, was further processed [17].

### 2.10. Culturing of Parasite

For cultivation process Hamid et al. (2012) method was used with some modifications. From the samples leishmanial parasite was cultured in the RPMI 1640 (Sigma, USA) culture media. The quantity of 0.3 g/30 mL of RPMI 1640 media was dissolved in (10 g/1000 mL) distilled water and added to 20 separate small screwed caped tubes. To avoid any bacterial and fungal contamination, the antibiotic Kanamycin was mixed. To the test tubes 1 mL of that sample was added in which amastigote was observed. At 25°C the test tubes were placed in incubator (Memmert type Inb 500, Germany). After 11 days of incubation period, promastigote of* Leishmania tropica* culture was observed with Giemsa staining and seen under Olympus Microscope at different magnifications 10x, 40x, and 100x [18].

### 2.11. Leishmanicidal Procedure

In laminar flow 4 mL of RPMI (1640) was taken in each test tube. About 20 *μ*L of leishmanial colony was added to each test tube along with positive control except for the test tube containing negative control. Then tubes were placed in incubator for 96 hours at 26°C. Then 1 mL from solutions of extracts and fractions of concentration 25 *μ*g/mL was added to these test tubes which contain media and shaken well for complete mixing. The activity of ethanolic extracts and other fractions was analyzed in nontreated control (DMSO without plant extracts) and other test tubes after time intervals of 24 hrs by microscope.

### 2.12. Phytochemical Analysis

Phytochemical tests were carried out on the ethanolic extract and other solvent soluble fractions using standard procedure to identify the constituents as described by Harborne and Sofowora for the detection of alkaloids, tannins, saponins, flavonoids, terpenes, and phenols.

*Alkaloids*. Dil. 70% HCl solution was mixed vigorously with extracts and fractions and then filtered. Hager's reagent (saturated solution of picric acid) was added to the filtrate. Yellow color precipitate formation shows the presence of alkaloids. 

*Saponins.* Extracts and fractions were separately heated with 10 mL of distilled water for 10 minutes. The mixture was filtered while being hot and was allowed to cool. Then 2.5 mL of filtrate was taken and 10 mL distilled water was added, and it was shaken vigorously for 2 minutes. Froth formation indicates the presence of saponins in the filtrate. 

*Tannins*. Extracts and fractions were separately heated with 20 mL of distilled water for five minutes in a water bath and were filtered while being hot. Then 1 mL of cool filtrate was mixed with 5 mL distilled water and a few drops (2-3) of 10% ferric chloride were added. A bluish-black or brownish-green precipitate indicates the presence of tannins. 

*Flavonoids. *Extracts and fractions were mixed with 10 mL of ethyl acetate, heated for few minutes on water bath, and then filtered. Mix 1 mL dil. ammonia solution with 4 mL filtrate. Yellow color precipitate formation shows the presence of flavonoids. 

*Terpenes.* Mix 0.5 mg of extract and fraction separately with 5 mL water and then add 3 to 4 drops of ethyl acetate. Formation of bright green color shows the presence of terpenes. 

*Phenols.* Add 2 to 3 drops of 0.1% FeCl_3_ solution to extracts and fractions separately. Formation of black or bluish color shows the presence of phenol [19, 20].

### 2.13. Analysis of Plant Samples for Heavy Metals

For dry digestion Khan et al. 2008 standard method was used with certain modifications. Precise weights of grinded and powdered plant sample of different parts were taken in China dish for heating in an oven at 105°C for several hours to confiscate moisture. This process is called charring. Then the moister free samples after charring were placed in furnace. The furnace temperature was gradually increased from room temperature to 550°C in 1 hr. The samples were ashed for about 5 hrs until a grey or white ash residue was obtained. The contents of China dish were cooled to room temperature in desiccators and 2 mL of 6 M HNO_3_ solution was added into China dish and, when required, the mixture was heated to dissolve its content. The solutions were filtered into 25 mL graduated cylinder and were diluted to the mark. The solutions were then stored in clean and dry plastic bottles. Estimation of heavy metals Fe, Zn, Ni, Co, Cu, Cr, Cd, Mn, and Pb was carried out on Flame Atomic Absorption Spectrophotometer (Perkin Elmer 400) [21].

## 3. Results and Discussion

### 3.1. Antibacterial Activity

*In vitro *antibacterial activities of crude extracts and other solvent soluble fractions of* Ballota nigra* were performed, and they were compared with the standard antibiotics (amoxicillin and levofloxacin).

In roots crude extracts show excellent inhibitory activity against all bacterial strains and have maximum inhibition against* Enterococcus faecalis* (24 mm),* Proteus mirabilis *(22 mm), and* Staphylococcus aureus* (21 mm). Chloroform fraction shows excellent inhibition results and maximum of 23 mm against* Proteus mirabilis.* Ethyl acetate shows promising activity against all bacterial strains and has maximum inhibition against* Staphylococcus aureus* (24 mm) and* Proteus mirabilis* (22 mm).

**Figure 1:**
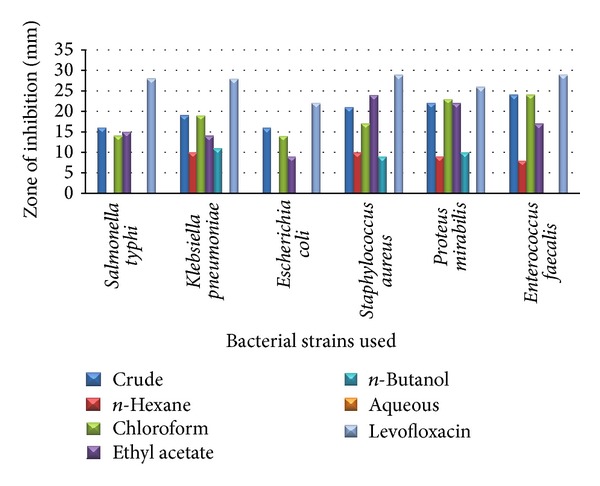
The antibacterial results of* B. nigra* root against different bacterial strains.

The antibacterial results of* B. nigra* root against different bacterial strains are given in Table 1 and Figure 1.

In stem ethanolic extract was active against all strains and aqueous fraction was inactive against all except* K. pneumoniae* and* E. coli.* Hexane fraction shows inhibitory activity against* S. aureus *and* E. coli.* Chloroform fraction was active against all and shows maximum inhibition (19 mm) against* S. aureus *and 18 mm against* P. mirabilis*. Ethyl acetate fraction shows inhibition against all apart from* E. coli.*

**Figure 2:**
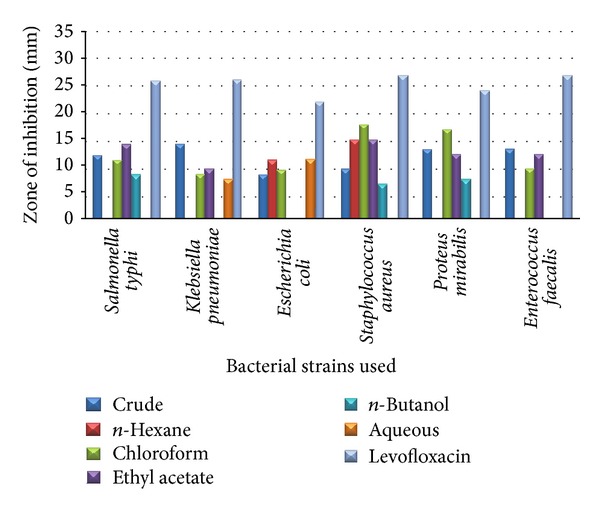
The antibacterial results of* B. nigra* stem against different bacterial strains.

The antibacterial results of* B. nigra* stem against different bacterial strains are shown in Table 2 and Figure 2.

In leaves all fractions with the exception of chloroform and butanol were inactive to* S. typhi*. Chloroform and butanol fraction show inhibition against all bacteria and maximum of 16 mm each against* K. pneumoniae*. Hexane fraction shows maximum activity of 18 mm against* K. pneumoniae.*

**Figure 3:**
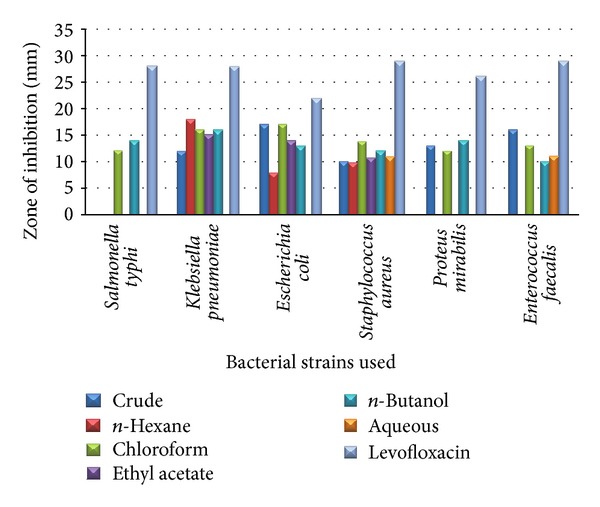
The antibacterial results of* B. nigra* leaves against different bacterial strains.

The antibacterial results of* B. nigra* leaves against different bacterial strains are shown in Table 3 and Figure 3.

### 3.2. Antifungal Activity

The antifungal results of* B. nigra *root, stem, and leaves are shown in Table 4.

*Aspergillus niger*. Crude extract and all fractions, except for aqueous fraction, were active and show inhibition in root, whereas crude ethanolic extract and hexane fraction were active fractions in stem. Ethyl acetate, aqueous, and* n-*hexane fractions in leaves were inactive to* A. niger.*

*Aspergillus fumigates. *All fractions except for hexane, aqueous, and* n-*butanol fractions were active in roots, while all fractions were active excluding butanol and aqueous fractions in stem and apart from aqueous and* n-*hexane fractions all were active in leaves against* A. fumigates*. 

*Aspergillus flavus.* All fractions not including aqueous fraction show activity in roots, whereas in stem crude, chloroform, and ethyl acetate fractions show inhibition and with the exception of aqueous and* n-*hexane fractions all were active in leaves against* A. flavus*. 

*Fusarium solani.* All fractions were inactive except crude and hexane in roots; all fractions except chloroform and aqueous were active in stem; and all fractions except aqueous show inhibition in leaves against* F. solani*. 

### 3.3. Antileishmanial Activity

Extracts and five fractions of different parts of* Ballota nigra* were analyzed for identification of active fractions against leishmanial parasite. Stock solutions of extracts and different fractions were made in DMSO at a concentration of 25 *μ*g/mL and 50 *μ*g/mL and were tested against leishmanial parasite.

Comparison of leishmanial parasite growth in media and DMSO control is shown in Table 5.

The antileishmanial results of different extracts and fractions of root stem and leaves were given in Tables 6, 7, and 8.

The results show that crude extract, chloroform, and ethyl acetate fractions of roots of* Ballota nigra* have leishmanicidal activity. Crude ethanolic extract, chloroform, and ethyl acetate fractions show inhibition from 6th day and do not convert amastigote form into active promastigote form.* n-*Hexane fraction shows inhibition from the 8th day, when amastigote form of leishmanial parasite was converted into active promastigote form. Aqueous and butanol fractions were inactive against amastigote. In stem the crude ethanolic extract starts inhibition from the 5th day; butanol and ethyl acetate fractions show inhibition of amastigote of leishmanial parasite from the 7th day. The* n-*hexane and chloroform fractions start inhibition of amastigote from the 8th day. The crude ethanolic extract, chloroform, and* n-*butanol fractions of leaves of* Ballota nigra* have leishmanicidal inhibitory action. Ethyl acetate fraction was only active in leaves and has good inhibition.* n*-Hexane fraction in all parts of the plant does not confirm any promising inhibitory activity.* n*-Hexane fraction gives inhibition after 7th day. It starts inhibition when amastigote life stage of leishmanial parasite was starting to convert into promastigote. Aqueous fraction was completely inactive in all parts of plant.

### 3.4. Phytochemical Analysis

In Table 9 results of phytochemical evaluation of different parts of different extracts and subfractions of* Ballota nigra *were shown.

The results show the phytochemical investigation of crude ethanolic extract and subsolvent soluble fractions of* B. nigra* roots. The crude extract and all fractions give negative result for alkaloids, saponins, and tannins. Flavonoids were present in ethanolic extract, chloroform, and ethyl acetate fractions. Terpenes were present in all fractions except* n-*butanol and aqueous fractions. Phenols give positive test in crude, chloroform, and ethyl acetate fractions.

In phytochemical investigation of crude ethanolic extract and fractions of* B. nigra* stem, alkaloids were absent in all fractions except for ethanolic extract. Flavonoids were present in crude, chloroform, and* n-*hexane fraction. Terpenes, tannins, and phenols were present in ethanolic extract, ethyl acetate, and* n-*butanol fractions. Saponins give negative test in all fractions.

In leaves of* Ballota nigra* saponins and tannins were absent in all fractions. Flavonoids and phenols were present in all fractions except in* n-*hexane and aqueous fractions. Terpenes were present in all fractions except in ethyl acetate and aqueous fractions.

### 3.5. Heavy Metals

The results obtained from atomic absorption spectrophotometer were then converted to meaningful data by the following formula, which gives us actual metal concentration in different parts of selected plants* Ballota nigra*:

(1) Concentration  of  metal  (mg/kg)=Observed  Conc.(ppm)×Vol.  of  Sample  Prepared (mL)Wt.  of  plant Sample (g).

**Figure 4:**
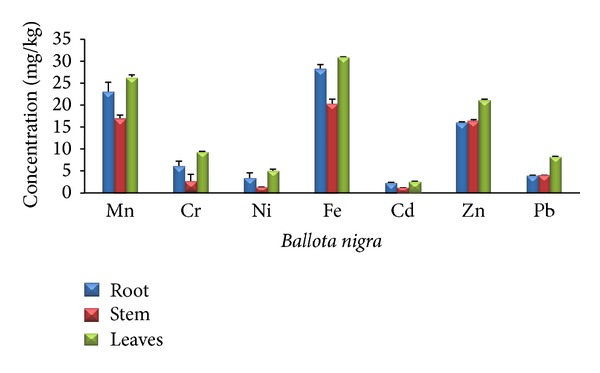
Concentration of different heavy metals in different parts (root, stem, and leaves) of* Ballota nigra *(mg/kg).

The concentration level of heavy metals in* Ballota nigra* root was found to decrease in the order of Fe > Mn > Zn > Cr > Pb > Ni > Cd. The concentration level of heavy metals in* Ballota nigra* stem was found to decrease in the order of Fe > Mn > Zn > Pb > Cr > Ni > Cd. The concentration level of heavy metals in* Ballota nigra* leaves was found to decrease in the order of Fe > Mn > Zn > Cr > Pb > Ni > Cd. The results are shown in Table 10 and Figure 4.

### 3.6. Heavy Metals above Permissible Limits

Table 10 and Figure 4 show that Chromium was above permissible value in all parts, whereas the WHO maximum permissible limit (MPL) of Chromium is 1.5 mg/kg [22]. Nickel was above WHO limit in* B. nigra* root and leaves, and the WHO permissible value of Nickel is 1.5 mg/kg [23]. Iron was above value in all parts of* B. nigra*; WHO MPL value of Iron is 20 mg/kg [22]. Cadmium was above permissible value in all parts and its MPL value is 0.3 mg/kg [22]. Lead was above permissible value in leaves while its WHO MPL value is 10 mg/kg [24].

## 4. Conclusion

The results of antimicrobial (antibacterial and antifungal) study of different parts of* Ballota nigra* indicate that the crude extract, ethyl acetate, and chloroform fractions were the most active fractions. The antiprotozoal (antileishmanial) activity of* Ballota nigra* was evaluated for the first time in which the crude extract, ethyl acetate, and chloroform fractions of* Ballota nigra* showed good leishmanicidal properties. But different parts have variable inhibition. It is clear from the results that plant* Ballota nigra* accumulates different phytochemicals in its different parts. These chemicals are responsible for various biological activities by disturbing the life cycle of different microbes and killing them. The results revealed that the plants accumulate different metals in their different parts (root, stem, and leaves) with diverse concentration. Only few metals (Cr, Ni, Fe, Cd, and Pb) were observed above WHO limits. So each medicinal plant should be analyzed for contaminant load or heavy metals before processing it for further pharmaceutical purposes or for local human consumption.

It is recommended that* Ballota nigra* is an important plant from a medicinal point of view and can be a potent candidate for further* in vivo* bioassays which would lead to the synthesis of safe herbal drugs with no or less side effects of global interest.

## Figures and Tables

**Table 1:** Antibacterial activity of *Ballota nigra *root.

Test microorganisms	Zone of inhibition (mm)													
	Crude		*n*-Hexane		Chloroform		Ethyl acetate		*n-*Butanol		Aqueous		Standards	
Crude extract/fractions (*μ*g/*μ*L)	2/1	2/1	2/1	2/1	2/1	2/1	2/1	2/1	2/1	2/1	2/1	2/1	Amx∗	Lev∗∗
*Salmonella typhi *	16	15	—	—	14	13	15	17	—	—	—	—	—	28
*Staphylococcus aureus *	20	21	10	10	17	17	23	24	09	08	—	—	—	29
*Proteus mirabilis *	22	22	09	11	23	22	22	21	08	10	—	—	—	26
*Klebsiella pneumoniae *	19	18	10	11	19	16	14	12	11	10	—	—	—	28
*Escherichia coli *	16	15	—	—	14	12	09	10	—	—	—	—	—	22
*Enterococcus faecalis *	24	23	08	09	24	22	17	16	—	—	—	—	—	29

(—): no inhibition zone (7 mm), ∗amoxicillin: 5 *μ*g/*μ*L, and ∗∗levofloxacin: 5 *μ*g/*μ*L.

**Table 2:** Antibacterial activity of *Ballota nigra *stem.

Test microorganisms	Zone of inhibition (mm)													
	Crude		*n*-Hexane		Chloroform		Ethyl acetate		*n-*Butanol		Aqueous		Standards	
Crude extract/fractions (*μ*g/*μ*L)	2/1	2/1	2/1	2/1	2/1	2/1	2/1	2/1	2/1	2/1	2/1	2/1	Amx∗	Lev∗∗
*Salmonella typhi *	11	13	—	—	12	12	15	15	08	09	—	—	—	28
*Staphylococcus aureus *	10	09	16	15	19	18	16	15	07	07	—	—	—	29
*Proteus mirabilis *	14	14	—	—	18	16	13	11	08	08	—	—	—	26
*Klebsiella pneumoniae *	15	13	—	—	09	09	10	10	—	—	08	07	—	28
*Escherichia coli *	08	09	12	12	10	09	—	—	—	—	10	12	—	22
*Enterococcus faecalis *	14	12	—	—	09	10	13	11	—	—	—	—	—	29

(—): no inhibition zone (7 mm), ∗amoxicillin: 5 *μ*g/*μ*L, and ∗∗levofloxacin: 5 *μ*g/*μ*L.

**Table 3:** Antibacterial activity of *Ballota nigra *leaves.

Test microorganisms	Zone of inhibition (mm)													
	Crude		*n*-Hexane		Chloroform		Ethyl acetate		*n-*Butanol		Aqueous		Standards	
Crude extract/fraction (*μ*g/*μ*L)	2/1	2/1	2/1	2/1	2/1	2/1	2/1	2/1	2/1	2/1	2/1	2/1	Amx∗	Lev∗∗
*Salmonella typhi *	—	—	—	—	11	12	—	—	13	14	—	—	—	28
*Staphylococcus aureus *	09	10	10	09	13	14	11	10	12	12	10	11	—	29
*Proteus mirabilis *	11	13	—	—	11	12	—	—	13	14	—	—	—	26
*Klebsiella pneumoniae *	12	11	18	17	16	16	14	15	16	16	—	—	—	28
*Escherichia coli *	17	16	08	08	17	16	13	14	13	11	—	—	—	22
*Enterococcus faecalis *	16	14	—	—	12	13	—	—	10	10	10	11	—	29

(—): no inhibition zone, ∗amoxicillin: 5 *μ*g/*μ*L, and ∗∗levofloxacin: 5 *μ*g/*μ*L.

**Table 4:** Antifungal activity of *Ballota nigra *(root, stem, and leaves).

	Fungal strains	Crude	*n-*Hexane	Chloroform	Ethyl acetate	*n-*Butanol	Water	Standard
								Clotrimazole∗
*Ballota nigra* (root)	*Aspergillus niger *	−	**−**	**−**	**−**	**−**	**+**	**−**
	*Aspergillus fumigatus *	−	**+**	**−**	**−**	**+**	**+**	**−**
	*Aspergillus flavus *	−	**−**	**−**	**−**	**+**	**+**	**−**
	*Fusarium solani *	−	**−**	**+**	**+**	**+**	**+**	**−**

*Ballota nigra* (stem)	*Aspergillus niger *	−	**−**	**+**	**+**	**+**	**+**	**−**
	*Aspergillus fumigatus *	−	**−**	**−**	**−**	**+**	**+**	**−**
	*Aspergillus flavus *	−	**+**	**−**	**−**	**+**	**+**	**−**
	*Fusarium solani *	−	**−**	**+**	**−**	**−**	**+**	**−**

*Ballota nigra* (leaves)	*Aspergillus niger *	−	**+**	**−**	**+**	**−**	**+**	**−**
	*Aspergillus fumigatus *	−	**+**	**−**	**−**	**−**	**+**	**−**
	*Aspergillus flavus *	−	**+**	**−**	**−**	**−**	**+**	**−**
	*Fusarium solani *	−	**−**	**−**	**−**	**−**	**+**	**−**

(−): inhibition, (**+**): no inhibition, and ∗Clotrimazole (12 *μ*g/6 *μ*L).

**Table 5:** *Leishmania* parasites in RPMI 1640 media and negative control (DMSO).

Media	Culture growth after days										
	1st	2nd	3rd	4th	5th	6th	7th	8th	9th	10th	11th
RPMI (1640)	−	−	−	−	**+ **	**+**	**++**	**++ **	**++ **	**++**	**++**
Negative control	−	−	−	−	−	−	−	−	−	−	−

(−) sign indicates amastigote form of cutaneous leishmaniasis; (+) indicates initial conversion of amastigote into promastigotes; and (++) shows promastigotes growth at peak level.

**Table 6:** Antileishmanial activity of *Ballota nigra* (root).

Extracts	Days 1-2	Day 3	Day 4	Day 5	Day 6	Day 7	Day 8	Day 9	Day 10	Day 11
Crude	+	+	+	+	−	−	−	−	−	−
*n-*Hexane	+	+	+	+	+	++	−	−	−	−
Chloroform	+	+	+	+	−	−	−	−	−	−
Ethyl acetate	+	+	+	+	−	−	−	−	−	−
*n-*Butanol	+	+	+	+	+	++	++	++	++	++
Aqueous	+	+	+	+	+	++	++	++	++	++

(−) indicates absence of amastigote and promastigote, (+) indicates presence of amastigote, and (++) indicates the presence of promastigote.

**Table 7:** Antileishmanial activity of *Ballota nigra* (stem).

Extracts	Day 1-2	Day 3	Day 4	Day 5	Day 6	Day 7	Day 8	Day 9	Day 10	Day 11
Crude	+	+	+	−	−	−	−	−	−	−
*n*-Hexane	+	+	+	+	+	++	−	−	−	−
Chloroform	+	+	+	−	−	−	−	−	−	−
Ethyl acetate	+	+	+	+	+	−	−	−	−	−
*n*-Butanol	+	+	+	+	−	−	−	−	−	−
Aqueous	+	+	+	+	+	++	++	++	++	++

(−) indicates absence of amastigote and promastigote, (+) indicates presence of amastigote, and (++) indicates the presence of promastigote.

**Table 8:** Antileishmanial activity of *Ballota nigra* (leaves).

Extracts	Days 1-2	Day 3	Day 4	Day 5	Day 6	Day 7	Day 8	Day 9	Day 10	Day 11
Crude	+	+	+	−	−	−	−	−	−	−
*n-*Hexane	+	+	+	+	+	++	−	−	−	−
Chloroform	+	+	+	−	−	−	−	−	−	−
Ethyl acetate	+	+	+	+	+	−	−	−	−	−
*n*-Butanol	+	+	+	+	−	−	−	−	−	−
Aqueous	+	+	+	+	+	++	++	++	++	++

(−) indicates absence of amastigote and promastigote, (+) indicates presence of amastigote, and (++) indicates the presence of promastigote.

**Table 9:** Phytochemical analysis of *Ballota nigra *(root, stem, and leaves).

Part used	Extracts	Alkaloids	Flavonoids	Terpenes	Tannins	Saponins	Phenols
*Ballota nigra* (root)	Crude	** −**	**+**	** +**	**−**	** −**	**+**
	*n-*Hexane	**−**	**−**	**+**	**−**	**−**	**−**
	Chloroform	**−**	**+**	**+**	**−**	**−**	**+**
	Ethyl Acetate	**−**	**+**	**+**	**−**	**−**	**+**
	Butanol	**−**	**−**	**−**	**−**	**−**	**−**
	Aqueous	**−**	**−**	**−**	**−**	**−**	**−**

*Ballota nigra* (stem)	Crude	**+**	**+**	**+**	**+**	**−**	**+**
	*n-*Hexane	**−**	**+**	**−**	**−**	**−**	**−**
	Chloroform	**−**	**+**	**−**	**−**	**−**	**−**
	Ethyl Acetate	**−**	**−**	**+**	**+**	**−**	**+**
	Butanol	**−**	**−**	**+**	**+**	**−**	**+**
	Aqueous	**−**	**−**	**−**	**−**	**−**	**−**

*Ballota nigra* (leaves)	Crude	**−**	**+**	**+**	**−**	**−**	**+**
	*n-*Hexane	**−**	**−**	**+**	**−**	**−**	**−**
	Chloroform	**−**	**+**	**+**	**−**	**−**	**+**
	Ethyl acetate	**−**	**+**	**−**	**−**	**−**	**+**
	Butanol	**−**	**+**	**+**	**−**	**−**	**+**
	Aqueous	**−**	**−**	**−**	**−**	**−**	**−**

(−) sign shows absence of phytochemicals and (+) sign shows presence of phytochemicals.

**Table 10:** Heavy metals concentration (mg/kg) in *Ballota nigra*.

Plant sample	Plant part used	Mn	Cr	Ni	Fe	Cd	Zn	Pb
*Ballota nigra *	Root	23.12 ± 2.13	06.04 ± 1.21	03.35 ± 1.20	28.20 ± 1.03	02.30 ± 0.07	16.08 ± 0.19	04.00 ± 0.02
	Stem	16.98 ± 0.76	2.74 ± 1.79	1.26 ± 0.06	20.34 ± 1.14	1.12 ± 0.05	16.35 ± 0.35	4.00 ± 0.026
	Leaves	26.42 ± 0.49	9.38 ± 0.07	5.09 ± 0.47	31.01 ± 0.09	2.53 ± 0.10	21.09 ± 0.22	9.25 ± 0.083
